# Dynamics in multiplicity of *Plasmodium falciparum* infection among children with asymptomatic malaria in central Ghana

**DOI:** 10.1186/s12863-017-0536-0

**Published:** 2017-07-17

**Authors:** Akua Kyerewaa Botwe, Kwaku Poku Asante, George Adjei, Samuel Assafuah, David Dosoo, Seth Owusu-Agyei

**Affiliations:** 1 Ghana Health Service. Health Research Unit, Kintampo Health Research Centre. MOH/GHS, P.O.Box 200, College of Health Street, Kintampo, Brong Ahafo Ghana; 2 Kwame Nkrumah University of Science and Technology, Kumasi, Ghana; 3 Department of Medicine, Solna, Karolinska Institutet, Stockholm, 17176 Sverige Sweden

**Keywords:** Asymptomatic, Falciparum, Malaria, MOI, Children, Kintampo, Ghana

## Abstract

**Background:**

The determinants of malaria parasite virulence is not entirely known, but the outcome of malaria infection (asymptomatic or symptomatic) has been associated with carriage of distinct parasite genotypes. Alleles considered important for erythrocyte invasion and selected as candidate targets for malaria vaccine development are increasingly being shown to have distinct characteristics in infection outcomes. Any unique/distinct patterns or alleles linked to infection outcome should be reproducible for a given malaria-cohort regardless of location, time or intervention. This study compared merozoite surface protein 2 (MSP2) genotypes from children with asymptomatic malaria at same geographical location, from two time periods.

**Results:**

As the prevalence and incidence of malaria (measured for other studies) significantly reduced between 2004 (time point one) and 2009 (time point two), MSP2 multiplicity of infections (MOI) also reduced significantly from 2.3 at time point (TP) one to 1.9 at TP two. IC/3D7 genotypes out-numbered FC27 genotypes at both time points. At TP2 however, FC27 allele diversity was more than the IC/3D7 allele diversity. A decrease in the IC/3D7:FC27 genotype proportions from 2:1 at TP1 to 1:1 at TP2, seemed to be driven mainly by a decrease in carriage of IC/3D7 alleles. MOI was higher in the dry season than in the subsequent wet season, but the decrease was not significant at TP2.

**Conclusion:**

MSP2 MOI was higher in the dry season than in the subsequent wet season, while the carriage of IC/3D7 alleles decreased over this time period. It may be that decreases in transmission are related specifically to the IC/3D7 allelic family. The influence of transmission on MSP2 allele diversity needs to be clearly deciphered in studies which should include the use of sensitive methods for the detection of polymorphic parasite markers for both symptomatic and asymptomatic malaria. Such studies will enable better understanding of associations between allelic variants, MOI, transmission, malaria infection and disease.

## Background

Reported estimates indicate a decrease in the number of people infected with malaria parasites from 131 million in 2010 to 114 million in 2015 and a 29% decrease in the number of malaria deaths in young children; nevertheless, malaria mortality remains high among children. In 2015, the infection rates were higher in children between 2 and 10 years of age than in other age groups, with 70% of the global total of deaths due to malaria estimated to have occurred in children below 5 years of age [1]. Malaria caused by *Plasmodium falciparum* was responsible for 99% of malaria deaths in 2015, and the disease ranges in severity from complicated, then mild (uncomplicated), to asymptomatic [1, 2]. In malaria-endemic areas, a significant proportion of children persistently harbour low levels of chronic parasitaemia in their blood stream without presenting any signs of clinical malaria and are considered asymptomatic cases [3, 4]. Severe (complicated) malaria is the principal cause of malaria related deaths, nevertheless asymptomatic malaria also plays an important role [2, 3, 5]. Asymptomatic malaria poses challenges for malaria control, it significantly influences parasite transmission dynamics and poses a public health danger to an entire population for as long as mosquito vector distribution and abundance remains [6]. Asymptomatic malaria can provide clues to understanding protection against clinical (symptomatic) malaria.

Asexual malaria parasites can be detected in individuals with either asymptomatic or symptomatic malaria, with the asymptomatic malaria individuals manifesting no malaria disease, whiles infected individuals with symptomatic malaria manifest malaria disease [6, 7]. The genetic characteristics of parasite populations detected (in children) during phases of asymptomatic carriage has been shown to differ from those causing a symptomatic malaria [8]. Although a number of determinants of parasite virulence has been proposed [9, 10], the considerable variation in parasite virulence has also been proposed to be possible by the absence of competition between the various parasite “strains” [7, 11]. The ability of *P. falciparum* to generate variability within its genetic families has been shown to be important in modulating the severity of malaria [7, 8, 10, 11]. This variability relies on the extensive allelic polymorphisms (known to be associated with resistance to antimalarial drugs), the antigenic variation and the sexual reproduction that ensures genetic mixing [12]. The number of different *P. falciparum* allelic variants of a given loci detected in an individual isolate (although additional alleles may easily remain unrecognized) represents the minimum number of the parasite genotypes, commonly referred to as the multiplicity of infection (MOI) [13]. MOI measures the number of parasite genotypes, which reflects recent exposure to infective mosquito bites [14].

The most effective and logistically feasible public health intervention against most infectious diseases is vaccination [15]. Rational development of vaccines is currently approached in two main ways: the sub-unit approach and the whole organism approach. Sub-unit vaccines can be made up of multiple or single antigens, to target the pre-erythrocytic, erythrocytic or sexual-stage antigens [15, 16]. One of several challenges posed to the rational development of sub-unit malaria vaccine is the determination of genetic variants of antigens and/or their functional mechanisms of protection during asymptomatic malaria [12, 17, 18]. Following vaccination with a genetically polymorphic target, it is important for non-target alleles to remain low. If the frequency of non-target alleles increases, the efficacy of the vaccine may be compromised [17]. Fortunately, there is evidence of associations between parasite alleles and malaria disease outcome [19–21], giving hope to the possibility of identifying frequenies of target alleles as well as important non-target alleles that play a role in malaria infection or disease pre and post vaccination.

Several studies have made it increasingly evident that parasite alleles considered important to erythrocyte invasion vary in malaria infection outcomes (symptomatic or asymptomatic) [22–25]. Asymptomatic carriage of parasites has been shown to be associated with reduced risk of symptomatic malaria [26, 27]. Much of the evidence seem to support the idea that the outcome of a malaria infection is associated with carriage of unique/variant parasite alleles. However, there is paucity of information regarding epidemiological trends for the independent outcome of infections or the combination of types or number of parasite alleles associated with the outcome of a malaria infection. Any unique/distinct patterns or alleles linked to infection outcome should be reproducible for a given malaria-cohort regardless of location, time or intervention. Studies which can determine the consistencies of unique characteristics in malaria infection outcome will contribute to the rational development of malaria vaccines. The determination of consistent pertinent characteristics of alleles could be important into setting a platform to argue for or against categorizing some alleles as either predominantly correlate markers for malaria infection or predominantly correlate markers for malaria disease. Such allele categorizations could have implications for selecting candidate vaccine targets.

The highly polymorphic merozoite surface protein 2 gene (MSP2) provides opportunity for such investigations. MSP2, previously known as MSA2, gp35–56, or GP3 [28, 29] play a role in the merozoite invasion of host erythrocytes and has been strongly associated with protection against malaria [26]. The several alleles of the MSP2 are grouped under 2 main families – the IC family and FC27 family. The difference between these two main allelic families are the dimorphic, non-repetitive sequences internal to the N- and C- termini [30, 31]. MSP2 remains a candidate antigen (having allele-specific effects) with potential for a subunit malaria vaccine amidst disappointing results from some trials [32]. In addition, in 2004 the MSP2 antigen was found to be the most polymorphic of the 3 polymorphic surface antigens (MSP1, MSP2 and Glutamate Rich Protein) analysed in the Kintampo area of Ghana where this study was conducted [33].

This study compared the MSP2 antigen diversity from children with asymptomatic malaria in 2003/2004 [Time point (TP1)] and 2009/2010 (TP2) to determine whether there are significant differences in parasite diversity and/or any consistently unique or distinct patterns for the polymorphic MSP2 antigen, at the same geographical location.

## Methods

### Study area

Analysis was carried out on samples collected from resident community members in the Kintampo North Municipality and the Kintampo South District at two time points using molecular biology facilities at the Kintampo Health Research Centre. The municipality and district lie within the forest savannah transitional ecological zone in the middle belt of Ghana. The Kintampo municipality and district cover an area of 7162 km2 and had a resident population of 134,970 in 2009 [34]. Mean monthly temperature ranges between 18 °C and 38 °C with average rainfall of 1250 mm per annum [33]. Up until 2003 when the first set of samples were taken for analysis (TP1 study), chloroquine was the first-line antimalarial drug in use for treatment of uncomplicated malaria in Ghana. Bed-net use among children less than 5 years old was 25.4% and the incidence of malaria in children below 5 years of age was 7 episodes per child per year [33]. Malaria transmission was high (entomological inoculation rate: 213 infective bites per person per year for the year of 2005) and perennial, peaking between April and October (major rainy season) and lowest from December to April (major dry season) of 2004 [35]. The drug policy changed in 2004 with artemisinin-based combination therapy (ACT) becoming first line antimalarial. In 2009 when the second set of samples were taken (TP2 study) for analysis, ACT use for the treatment of uncomplicated malaria in Ghana/Kintampo was virtually universal. Bed-net use had increased from 25.4% (measured for children) to 47.4% (measured for pregnant women), and the incidence of malaria in children below 5 years of age, had decreased from 7 to 1.3 episodes per child per year [36].

### Study design

The TP1 study enrolled 1113 children [33], while the TP2 study enrolled 1855 children [36]. The TP1 MSP2 MOI and allelic distributions, previously described [4] was obtained by sampling from the study area sixteen “index cluster” of contiguous houses, randomly selected from a total of about 22,000 houses, bimonthly for a year [33]. The random selection of compounds from each of the 16 “index clusters” ensured as much as possible adequate representation of micro-ecological factors such as water bodies and vegetation types that are known to influence malaria transmission. A minimum number of 140 consented participants were selected to represent each cluster. A total of 308 blood blot filter papers, pre-determined as positive by microscopy, from children with asymptomatic malaria and below 5 years of age were sampled ensuring that each of the 16 clusters were represented in the six bi-monthly cross-sectional surveys at TP1. MSP2 genotyping was carried out for all 308 infant samples. Similarly, blood blot filter papers from 310 children aged below 3 years with asymptomatic malaria were selected from the same study area. These (TP2 study samples) were selected via six bi-monthly cross-sectional sampling and were parasite positive by microscopy. The molecular studies we describe, used blood blot filter paper samples from children in the TP1 and TP2 studies, thus no child participated in both the TP1 or TP2 molecular studies. The MOI results from TP1 study were compared with MOI results from TP2 study. A child was classified as asymptomatic if clinical symptoms or fever was absent (i.e. axillary temperature < 37.5 °C) but *P. falciparum* parasites present by microscopy. All participants whose data are included in this analyses provided informed consent/assent. The genotyping of MSP2 (block 3) was performed on samples from the TP2 study as described below.

### Extraction of genetic material (DNA)

Total deoxy-ribonucleic acid (DNA) was extracted from dried blood spots on filter paper using the Qiagen (QIAamp DNA mini kit) DNA extraction protocol for DNA extraction from filter paper. The manufacturer’s protocol was followed. The DNA of the MSP2 genotype was amplified by polymerase chain reactions.

### Polymerase chain reaction

Nested polymerase chain reactions (PCR) were carried out to optimise primers for the FC27 and IC variants of the MSP2 (Nest 1 *forward primer*: 5′**-**ATG AAG GTA ATT AAA ACA TTG TCT ATT ATA, *reverse primer*: 5′-CTT TGT TAC CAT CGG TAC ATT CTT. Nest 2 *IC forward primer*: 5′-AGA AGT ATG GCA GAA AGT AAK CCT YCT ACT, *IC reverse primer*: 5′-GAT TGT AAT TCG GGG GAT TCA GTT TGT TCG, *FC forward primer*: 5′-AAT ACT AAG AGT GTA GGT GCA RAT GCT CCA, *FC reverse primer*: 5′-TTT TAT TTG GTG CAT TGC CAG AAC TTG AAC). The analyses was carried out based on procedures previously described [13]. The nest 1 and 2 PCR procedures which were used are outlined. A total reaction volume of 25 μl was used for the nest 1 amplifications. The nest 1 reaction consisted of 1× Qiagen buffer (containing 15 mM MgCl_2_), 1.5 mM MgCl_2_, 0.50 mM dNTP, 0.4 mM oligonucleotide primers, 1.0 U of Qiagen Taq polymerase, and 2 μl template. The PCR was performed at an initial denaturation of DNA at 95 °C for 3 min, and a latter denaturation at 95 °C for 45 s. Following the denaturation, 29 cycles of primer annealing at 57 °C for 45 s, and DNA extension at 72 °C for 1 min were carried out. The final primer extension after the cycles were carried out at 72 °C for 1 min, and the reaction cooled to 12 °C.

The nest 2 total reaction volume was 25 μl. This reaction contained 1× Qiagen buffer (containing 15 mM MgCl_2_), 1.5 mM MgCl_2_, 0.50 mM dNTPs, 0.4 mM oligonucleotide primers, 1.0 U of Qiagen Taq. polymerase, and 2 μl template. The PCR was performed with an initial denaturation of DNA at 95 °C for 3 min followed by 29 cycles of denaturation at 95 °C for 45 s, primer annealing at 59 °C for 30 s and 1 min of DNA extension at 72 °C. The final extension was at 72 °C for 10 min and samples cooled to 12 °C until it was removed from the thermal cycler.

The nest 2 amplification products were analysed by agarose gel electrophoresis; using 5 μl of the amplified reaction product in a 2% agarose gel, containing 0.4-mg/ml ethidium bromide. A 100 base pair molecular weight marker/standard was added to the electrophoresis to evaluate the sizes of amplified PCR products (DNA). Visualisation of the different sized DNA was performed with an ultraviolet light transilluminator connected to a desktop computer. The different sizes of DNA fragments (bands) seen were recorded. The total number of bands (parasite genotypes) from an individual isolate represented an estimate of the MSP2 MOI for the individual. The mean MOI for MSP2 allelic families (FC27 or IC/3D7) was determined as the sum of parasite genotypes from the family, divided by the total number of hosts with positive PCR samples (hosts with carriage of alleles from the MSP2 allelic family). The sum of parasite genotypes at a given time point, divided by the total number of hosts with positive PCR samples at the time point, represented an estimate of the mean MSP2 MOI at that time point [4, 37]. Resulting genotypes were grouped on a bi-monthly (Nov/Dec etc.) basis for the entire year. The bi-monthly MOI for TP1 study (calculated as described above) was compared with bi-monthly MOI results obtained for TP2 study. Also, the MOI of the dry season and the MOI of the wet season were calculated from the resulting genotypes identified from November to April and from May to October respectively.

### Quality control of PCR

Standard operating procedures and good clinical laboratory practices were followed throughout the analyses. Two negative controls consisting of only reagents (no template controls), one generated during DNA extraction and the other at the PCR stage, were added to all reactions. Positive *P. falciparum* controls (IC/3D7: 450 base pairs and HB3: 280 base pairs) were sourced from Malaria Research Reference Reagent Resource Centre. The samples analyzed by agarose gel electrophoresis included a standard base-pair marker. If there was no amplification, the electrophoresis was repeated using twice of the quantity of the PCR product. When the repeated electrophoresis did not show amplification, the PCRs were repeated using twice of the DNA quantity. Where non-amplifications persisted after this second PCR, the amplification was classified as unsuccessful. *P. falciparum* species identification was carried out for unsuccessful amplifications. Unaltered-images of gel electrophoresed PCR products were scored independently by two molecular biologists. In the event of conflicting scores, a third independent molecular biologist also scored. Same methods were used in the TP1 analyses [4].

### Statistical analysis

The non-parametric Kruskal-Wallis test was used to compare the bi-monthly arithmetic mean MOI results of TP2 only for significant differences between at least a pair of means. The Dunn’s pairwise test was further used with the Bonferroni adjustment method to detect pairwise differences in means. Statistical comparison of TP1 and TP2 MOI were also made using the Wilcoxon rank-sum test (overall and bi-monthly). All p– values which were less than 0.05 were statistically significant. STATA version 14.0 software was used for the statistical analysis.

## Results

A total of 243 successful PCR amplifications were obtained from 308 samples analysed in the TP1 study [4], while a total of 136 successful PCR amplifications were obtained from 310 samples analysed at TP2. Samples which were successfully amplified for analyses in November/December 2009 (TP2) were 36, while in January/February, March/April, May/June, July/August, September/October of 2010 (TP2) the successful amplifications were 25, 29, 13, 13 and 16 respectively. Successful amplifications for TP1 have previously been reported [4]. Carriage of 7 genotypes (highest MOI) was among two children from TP1 and one child from TP2, all observed in the dry season. Carriage of a single parasite clone (most frequent) was among 88 children from TP1 (46 children in dry season, and 42 children in wet season) and 60 children from TP2 (39 children in the dry season and 21 in the wet season).

TP1 participants were aged below 5 years, while TP2 participants were aged below 3 years. Comparing the mean MOI (2.2) for children below age 3 with the mean MOI (2.3) for children aged between 3 to 5 (<5) years at TP1, no statistically significant differences (*P* = 0.56) in mean MOI was found.

At TP2, the Kruskal Wallis test indicated statistically significant difference in mean MOI for at least 2 of the months. The Wilcoxon’s rank sum test showed that the mean MOI reduced from 2.3 (SD = 1.3) at TP1 to 1.9 (SD = 1.2) at TP2, and this was statistically significant (*P* = 0.021). Table 1 shows the significance of the differences in mean MOIs for the various months.

**Table 1:** MSP2 mean MOI at TP1 and TP2

Months	TP1			TP2			
	Parasite Genotypes	Mean MOI (^a^SD)	Range	Parasite Genotypes	Mean MOI (SD)	Range	*P*-values
Nov/Dec	106	1.9 (0.9)	1–4	96	2.5 (1.6)	1–7	0.07
Jan/Feb	124	2.4 (1.4)	1–7	24	1.2 (0.5)	1–2	0.001
Mar/Apr	127	3.2 (1.5)	1–7	57	2.0 (1.0)	1–4	< 0.001
May/Jun	68	2.1 (1.2)	1–5	20	1.5 (0.8)	1–3	0.123
Jul/Aug	88	2.3 (1.3)	1–6	25	1.9 (1.0)	1–4	0.426
Sept/Oct	37	1.4 (0.9)	1–4	28	1.7 (0.9)	1–3	0.213
Total	550	2.3 (1.3)	1–7	(250)	1.9 (1.2)	1–7	0.021

^a^ *SD* standard deviation

### Distribution of MSP2 diversity by season of year

**Fig. 1:**
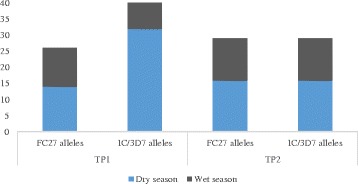
MSP2 allele diversity for TP1 and TP2

**Fig. 2:**
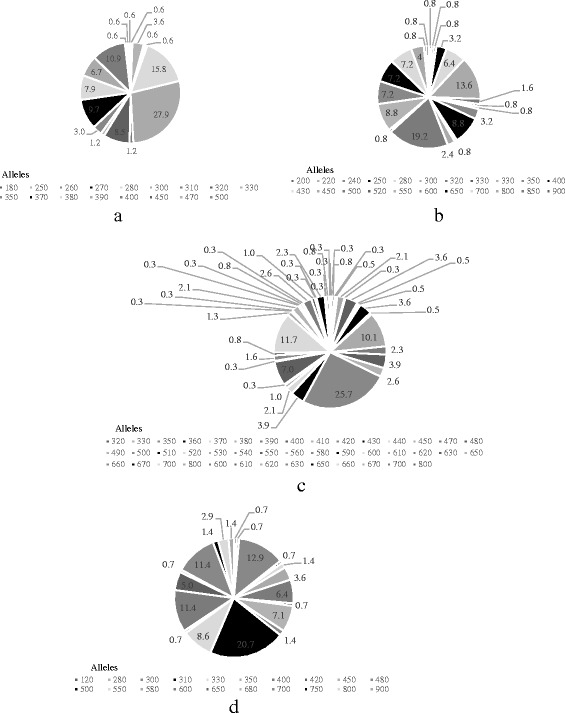
MSP2 allelic frequencies at TP1 and TP2. 2a and 2b shows the FC27 allelic frequencies at TP1 and TP2 respectively, while 2c and 2d shows the IC/3D7 allelic frequencies at TP1 and TP2 respectively

A comparison of the MSP2 mean MOIs of the two seasons in Table 2, showed reduction in MOI from the dry season to the wet season at both TP1 and TP2. This reduction was significant (*p* = 0.025) at TP1 but not significant at TP2 (*p* = 0.348). During the dry season of TP1, 32 different IC/3D7 alleles (320 bp to 500 bp) with a predominant 500 bp allele were identified, while 14 different FC27 alleles (250 bp – 450 bp) with a predominant 300 pb allele were identified (Figs. 1 and 2). During the wet season at TP1 twenty-one IC/3D7 alleles, (330 - 700 bp) with a predominant 500 bp allele were identified, while twelve FC27 alleles (180 – 500 bp) with a predominant 300 pb allele were identified (Figs. 1 and 2). At TP2 sixteen IC/3D7 alleles (280 – 900 bp) with a predominant 500 bp allele, and sixteen FC27 alleles (250 – 850 bp) with a predominant 500 pb allele were identified during the dry season (Figs. 1 and 2). Also in the wet season, thirteen IC/3D7 alleles (120 – 900 bp) with a predominant 500 bp, and thirteen FC27 alleles (200 – 900 bp), with a predominant 300 bp and 400 pb were identified (Figs. 1 and 2).

**Table 2:** Seasonal MSP2 MOI for TP1 and TP2

Season of year	TP1 mean MOI (SD)	TP2 mean MOI (SD)
Dry Season (Nov to Apr)	2.4 (1.4)	2.0 (1.3)
Wet Season (May to Oct)	2.0 (1.2)	1.7 (0.9)
*P*-values	0.025	0.348

### Impact of time on MSP2 diversity

The MSP2 IC/3D7 genotypes were detected more frequently than FC27 genotypes at both TP1 and TP2 (Table 3). However, whereas the proportion of FC27: IC/3D7 genotypes was 1:2 at TP1, the proportion of FC27: IC/3D7 genotypes was 1:1 at TP2 (Table 3). The MSP2 allele fragments were 60 at TP1 and 42 at TP2 (Table 3). At TP1, the IC/3D7 allele diversity (43 alleles) was higher than the FC27 allele diversity (17 alleles), and ranged between 320 bp – 800 bp and 180 bp – 500 bp respectively (Table 3 and Fig. 2). The predominant alleles for the IC/3D7 and FC27 allelic families were 500 bp (25.7%) and 300 bp (27.9%) respectively at TP1 (Fig. 2). At TP2 the FC27 allele diversity (22 alleles) was higher than the IC/3D7 allele diversity (20) and ranged between 200 bp – 900 bp and 120 bp – 900 bp respectively (Table 3 and Fig. 2). The predominant alleles for the IC/3D7 and FC27 allelic families were 500 bp (20.7%) and 500 bp (19.2%) at TP2 (Fig. 2). FC27 alleles 350 bp and 330 bp and IC/3D7 allele 420 bp were present in the dry seasons but absent in the wet seasons (transient) at both TP1 and TP2.

**Table 3:** Proportions of MSP2 genotypes and alleles at TP1 and TP2

	TP1		TP2	
Allelic family	FC27	IC/3D7	FC27	IC/3D7
Parasite genotypes	165	385	125	140
Proportion of genotypes	0.3	0.7	0.5	0.5
Number of alleles	17	43	22	20

## Discussion

There is paucity of information regarding epidemiological trends for the independent outcome of malaria infections or the combination of types or number of parasite alleles associated with outcome of infection. Asymptomatic and symptomatic outcomes of malaria infections have been associated with carriage of distinct parasite genotypes. Alleles considered important for erythrocyte invasion and selected as candidate targets for malaria vaccine development are increasingly being shown to have distinct characteristics in infection outcomes. Any unique/distinct patterns of alleles linked to infection outcome should be reproducible for a given asymptomatic or symptomatic malaria-cohort regardless of location, time or intervention. Therefore, we conducted this study to determine whether MSP2 diversity from children with asymptomatic malaria has pertinent characteristics in time at the same geographical location.

The results of this study showed significant reduction in MSP2 MOI from 2.3 in 2004 (TP1) to 1.9 in 2009 (TP2), within a half-decade among children under 5 years of age who developed asymptomatic malaria. At time point 1 (TP1) and time point 2 (TP2), the MSP2 IC/3D7 genotypes out-numbered the FC27 genotypes. Variations in the IC/3D7:FC27 proportions 2:1 at TP1 and 1:1 at TP2 was observed. The FC27 allele diversity was higher at TP2 (22 alleles) than at TP1 (17 alleles). Also at TP2, FC27 allele diversity (22 alleles) was higher than IC3D7 allele diversity (20 alleles). Based on the overall number of alleles and genotypes observed, the variations in genotype proportions appears to be primarily driven by a decrease in the carriage of IC/3D7 alleles. A year to year variation in malaria epidemiology/transmission (measured by parasite prevalence) would be gradual [38], hence the parasite genetic epidemiology. Thus, these differences in allele diversity may be reflective of cumulative variations (within 4 years’ interval from TP1 to TP2), which may be partly due to malaria interventions such as increased use of ITNs and ACTs in Kintampo [33, 36, 38]. Additional studies to compare symptomatic and asymptomatic malaria is required to confirm selectivity of alleles over time and relationships between decreasing transmission and allelic diversity.

The debate that high MOI in early life is necessary to reduce risk of developing clinical malaria is ongoing [14, 39, 40]. The significant reduction in MOI from 2.3 at TP1 to 1.9 at TP2, with a corresponding decrease in the proportions and number of IC/3D7 genotypes and alleles, among this asymptomatic cohort may suggest that specific allele variants with/without high MOI may be involved in a malaria infection outcome. Again, additional studies using sensitive methods for the detection of minor alleles are required, to clearly determine relationships between asymptomatic malaria and allelic variations.

There were more single-clone and multi-clone parasite infections in the dry than in the wet season at both time points. Measures put in-place to minimize and avoid template conditions included: use of single template PCRs, use of a fresh aliquot of primers whenever non-specific amplifications was detected, paying attention to negative controls and being mindful of contamination. The higher MOIs in the dry season compared with the wet season is consistent with studies among infants in the Ashanti and Northern Regions of Ghana. These studies detected more infections in the rainy than in the dry season but unexpectedly higher MSP diversity in the dry season than in the rainy season [41] and lowered MOI in the rainy season with higher polyclonal infections in the dry seasons [42]_._ In South-Western Cameroon where malaria transmission is perennial, a similar paradox was observed where infants born during the wet season were more susceptible to malaria infection while children born in the dry season were protected [43]. MOIs for residents of Kintampo coincided with the lowest entomological inoculation rates (EIR), at the end of the dry season, while the lowest MOIs coincided with the highest EIR at the end of the wet season [4, 35]. In this study EIR was not measured, however MOI appears to be high in the dry (low transmission) season and low in the wet (high transmission) season. Also, some transient MSP2 alleles which were present in the dry season were absent during the subsequent wet season, while other alleles were present in both seasons. In this study, MSP2 alleles presented during symptomatic malaria were not tracked, nevertheless, the essence of carriage of some alleles throughout both wet and dry seasons may be as explained by studies carried out by Sonden et al. (2015) [27] in a high transmission area of Mali. Their results suggested that “persistent multiclonal infections carried through the dry season contribute to protection against subsequent febrile malaria possibly by maintaining protective immune responses that depend on ongoing parasite infection” [27]. These observations are worth noting for a substantive explanation for increased parasite genotypes during low transmission seasons, compared to the high transmission season. As a marker of exposure to malaria parasites [14], the MOI can reveal patterns in malaria transmission. Therefore studies to determine the effect of factors such as vectors and climate (rainfall and temperature which can influence changes in malaria transmission [44]) on patterns of MOI in the dry and wet seasons may be necessary. Any climatic impacts on MOI may have important implications (with respect to immune correlates) for the conduct of some malaria vaccine trials.

Unlike at TP2 where the Qiagen method was used for DNA extraction, at TP1 the chelex method was used. The amounts of DNA extracted at TP1 and TP2 could not be compared quantitatively, and so the ability of one method to extract DNA more efficiently than the other, and subsequently introduce bias could not be determined. Nevertheless no significant differences between results obtained from the use of these two methods have been identified, using both buccal cells and blood stains as the DNA source [45]. Therefore, the change in method of extracting the DNA at TP2 is not perceived to bias results. All methods used were comparable. Electrophoretic variability between TP1 and TP2 during the establishment of allelic frequencies cannot be overlooked. The ability to precisely determine the size of an allele ultimately depends on the method of resolution of amplified DNA fragments. At present, capillary gel electrophoresis will be recommended as a reliable method for accurate sizing of alleles, because it is more sensitive for detection of accurate DNA fragment lengths relative to agarose gel electrophoresis.

## Conclusion

Findings from this study showed significant reduction in MSP2 MOI, with a corresponding decrease in the carriage of IC/3D7 genotypes and allele diversity, within a half-decade at Kintampo. MSP2 allele diversity was observed in the asymptomatic status but in different proportions at both time points. A decrease in the IC/3D7:FC27 proportions from 2:1 at TP1 to 1:1 at TP2, driven mainly by a decrease in carriage of IC/3D7 alleles was observed. Three MSP2 allele variants appeared to be transient, while other alleles persisted throughout both dry and wet seasons. Also, MSP2 MOI was higher during the low transmission season than the subsequent high transmission season at TP1 and TP2. MSP2 allele diversity appeared to be influenced in a transmission-dependent manner. It may be that decreases in transmission are related specifically to the IC/3D7 allelic family. To clearly decipher relationships between transmission and alleles will require additional studies, which should employ the use of sensitive methods and measurements for the detection of polymorphic parasite markers for both symptomatic and asymptomatic malaria.

## Abbreviations

DNA: Deoxy-ribonucleic acid
EIR: Entomological inoculation rate
MOI: Multiplicity of infection
MSP: Merozoite surface protein
PCR: Polymerase chain reaction
TP: Time point
